# Population Pharmacokinetics and Cerebrospinal Fluid Penetration of Fluconazole in Adults with Cryptococcal Meningitis

**DOI:** 10.1128/AAC.00885-18

**Published:** 2018-08-27

**Authors:** Katharine E. Stott, Justin Beardsley, Ruwanthi Kolamunnage-Dona, Anahi Santoyo Castelazo, Freddie Mukasa Kibengo, Nguyen Thi Hoang Mai, Nguyễn Lê Nhu’ Tùng, Ngo Thi Kim Cuc, Jeremy Day, William Hope

**Affiliations:** a Antimicrobial Pharmacodynamics and Therapeutics Laboratory, Department of Molecular and Clinical Pharmacology, Institute of Translational Medicine, University of Liverpool, Liverpool, United Kingdom; b Malawi-Liverpool-Wellcome Trust Clinical Research Programme, Blantyre, Malawi; c Oxford University Clinical Research Unit, Ho Chi Minh City, Vietnam; d Department of Biostatistics, Institute of Translational Medicine, University of Liverpool, Liverpool, United Kingdom; e MRC/UVRI Uganda Research Unit on AIDS, Entebbe, Uganda; f Hospital for Tropical Diseases, Ho Chi Minh City, Vietnam; g Centre for Tropical Medicine and Global Health, Nuffield Department of Medicine, University of Oxford, Oxford, United Kingdom

**Keywords:** cryptococcal meningitis, pharmacokinetics, pharmacodynamics, fluconazole, central nervous system pharmacokinetics, central nervous system infections, meta-analysis

## Abstract

Robust population pharmacokinetic (PK) data for fluconazole are scarce. The variability of fluconazole penetration into the central nervous system (CNS) is not known. A fluconazole PK study was conducted in 43 patients receiving oral fluconazole (usually 800 mg every 24 h [q24h]) in combination with amphotericin B deoxycholate (1 mg/kg q24h) for cryptococcal meningitis (CM). A four-compartment PK model was developed, and Monte Carlo simulations were performed for a range of fluconazole dosages. A meta-analysis of trials reporting outcomes of CM patients treated with fluconazole monotherapy was performed. Adjusted for bioavailability, the PK parameter means (standard deviation) were the following: clearance, 0.72 (0.24) liters/h; volume of the central compartment, 18.07 (6.31) liters; volume of the CNS compartment, 32.07 (17.60) liters; first-order rate constant from the central to peripheral compartment, 12.20 (11.17) h^−1^, from the peripheral to central compartment, 18.10 (8.25) h^−1^, from the central to CNS compartment, 35.43 (13.74) h^−1^, and from the CNS to central the compartment, 28.63 (10.03) h^−1^. Simulations of the area under concentration-time curve resulted in median (interquartile range) values of 1,143.2 (range, 988.4 to 1,378.0) mg · h/liter in plasma (AUC_plasma_) and 982.9 (range, 781.0 to 1,185.9) mg · h/liter in cerebrospinal fluid (AUC_CSF_) after a dosage of 1,200 mg q24h. The mean simulated ratio of AUC_CSF_/AUC_plasma_ was 0.89 (standard deviation [SD], 0.44). The recommended dosage of fluconazole for CM induction therapy fails to attain the pharmacodynamic (PD) target in respect to the wild-type MIC distribution for C. neoformans. The meta-analysis suggested modest improvements in both CSF sterility and mortality outcomes with escalating dosage. This study provides the pharmacodynamic rationale for the long-recognized fact that fluconazole monotherapy is an inadequate induction regimen for CM.

## Abstract (precis)

Robust population pharmacokinetic (PK) data for fluconazole are scarce. The variability of fluconazole penetration into the central nervous system (CNS) is not known.

## INTRODUCTION

Mortality from cryptococcal meningitis remains unacceptably high. More than 90% of the estimated 223,100 annual incident cases of cryptococcal meningitis occur in Sub-Saharan Africa and Asia-Pacific regions (1). The most effective regimen for induction is amphotericin B deoxycholate and flucytosine (2, 3). However, access to these drugs is limited in many regions where the burden of cryptococcal meningitis is greatest (4, 5). In these settings, high-dose fluconazole is used for induction monotherapy, despite consistent evidence of reduced survival in comparison to that with other agents and combinations (6–8).

Fluconazole was discovered by Pfizer, Inc. (Sandwich, United Kingdom) in 1978 (9). The objective was to discover an orally bioavailable agent for the treatment of invasive mycoses with a lower propensity to develop resistance than flucytosine (9). Fluconazole inhibits cytochrome P450-dependent demethylation of lanosterol in the ergosterol biosynthetic pathway (10). The ratio of the area under the concentration-time curve (AUC) to the MIC is the pharmacodynamic (PD) index that best links drug exposure of fluconazole with the observed antifungal effect (11, 12).

Successful antimicrobial therapy within the central nervous system depends on the achievement of effective drug concentrations within relevant subcompartments that include the cerebrum, meninges, and cerebrospinal fluid (CSF) (13). Fluconazole has a low molecular weight (approximately 300 g/mol), is weakly protein bound, and is not known to be a substrate for central nervous system (CNS) efflux pumps (14, 15). Its ability to partition from the endovascular compartment into the CNS has been established in laboratory animal models (16, 17) and clinical studies (18, 19). Brain/plasma penetration ratios of up to 1.33 have been reported in humans (19). However, there is a surprising paucity of population pharmacokinetic (PK) data for fluconazole in all clinical contexts. Furthermore, the extent and variability of penetration into the CNS are not known.

The primary aim of this study was to quantify the extent and variability of CNS penetration of fluconazole in adults with cryptococcal meningitis. We developed a population PK model that quantified the interindividual variability in drug exposure in plasma and cerebrospinal fluid (CSF). We investigated the impact of a range of clinically relevant covariates on fluconazole PK. Monte Carlo simulation was used to assess the implications of PK variability in terms of achieving fluconazole PD targets. Finally, we conducted a meta-analysis of clinical trials of fluconazole monotherapy to estimate the contribution of dosage to clinical outcome.

## RESULTS

### Patients.

A total of 43 patients (23 from Vietnam and 20 from Uganda) were recruited over an 11-month period between January and November 2016. Twenty-two patients (52%) were female. Patient characteristics (overall median [range]) were the following: age, 33 years (20 to 73 years); weight, 48 kg (32 to 68 kg); body mass index (BMI), 18 kg/m^2^ (12 to 25 kg/m^2^); creatinine at enrollment, 70 μmol/liter (37 to 167 μmol/liter); and estimated glomerular filtration rate (eGFR) using the Cockcroft-Gault equation, 84.8 ml/min/1.73 m^2^ (35.4 to 146.7 ml/min/1.73 m^2^). The baseline creatinine concentration was significantly lower in Vietnamese patients than in Ugandan patients (median, 56 versus 79 μmol/liter; *P* value, 0.02). However, this did not manifest as a significant difference in eGFR due to different age, sex, and weight profiles between the two patient populations. There were no statistically significant differences between ethnic groups for other demographic variables. The demographic data are shown by ethnicity and for the study population as a whole in Table 1.

**TABLE 1:** Patient demographics

Demographic or clinical characteristic^*a*^	Value for the group			*P* value^*g*^
	Vietnam	Uganda	Combined	
Sex^*b*^				
No. of males	13	8	23	
No. of females	10	12	20	
Age (yr)^*c*^				0.75
Mean	38	33	35	
Median	33	33	33	
Range	20–73	24–50	20–73	
Weight (kg)^*d*^				0.23
Mean	46	49	48	
Median	45	49	48	
Range	32–68	35–60	32–68	
BMI (kg/m^2^)^*e*^				0.73
Mean	18	18	18	
Median	18	18	18	
Range	12–25	15–22	12–25	
Creatinine (μmol/liter)^*b*^				0.02
Mean	67	81	74	
Median	56	79	70	
Range	37–167	43–145	37–167	
eGFR (ml/min/1.73 m^2^)^*f*^				0.10
Mean	88.3	80.7	84.7	
Median	84.8	81.4	84.8	
Range	35.4–136.1	49.8–146.7	35.4–146.7	

a BMI, body mass index; eGFR, estimated glomerular filtration rate, by Cockcroft-Gault equation.

b *n* = 43.

c *n* = 31.

d *n* = 41.

e *n* = 35.

f *n* = 33.

g *P* value for difference between Vietnam and Uganda groups by Mann-Whitney test of significance.

### Pharmacokinetic data.

**FIG 1:**
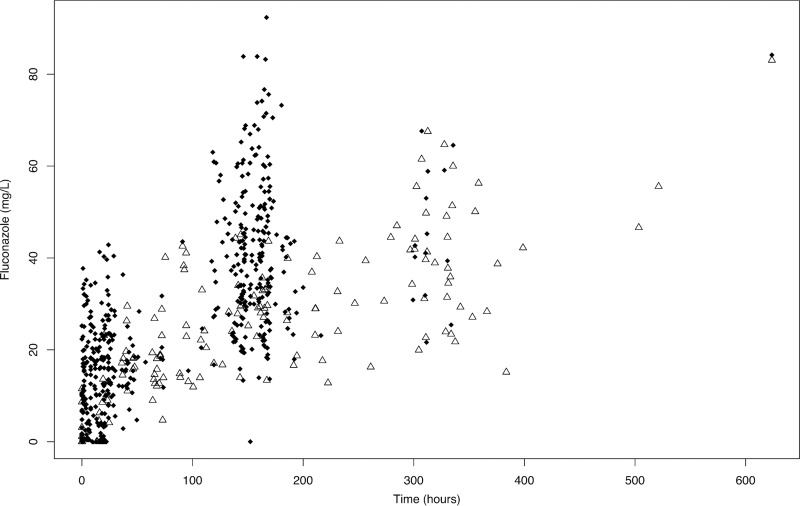
Fluconazole concentrations in 43 patients. Black diamonds represent plasma concentrations. White triangles represent CSF concentrations.

The final data set included 312 plasma observations and 52 CSF observations from the Vietnamese cohort. From the Ugandan cohort, the data set included 196 plasma observations and 115 CSF observations. A single CSF observation from one Ugandan patient was excluded because no fluconazole was detectable in an isolated sample after 13 days of therapy. This was inconsistent with results from other patients and could not be verified. The mean numbers of plasma samples and CSF samples per patient were 11.8 and 3.9, respectively. Figure 1 shows the raw plasma and CSF concentration-time profiles from study participants.

### Population pharmacokinetic analysis.

**FIG 2:**
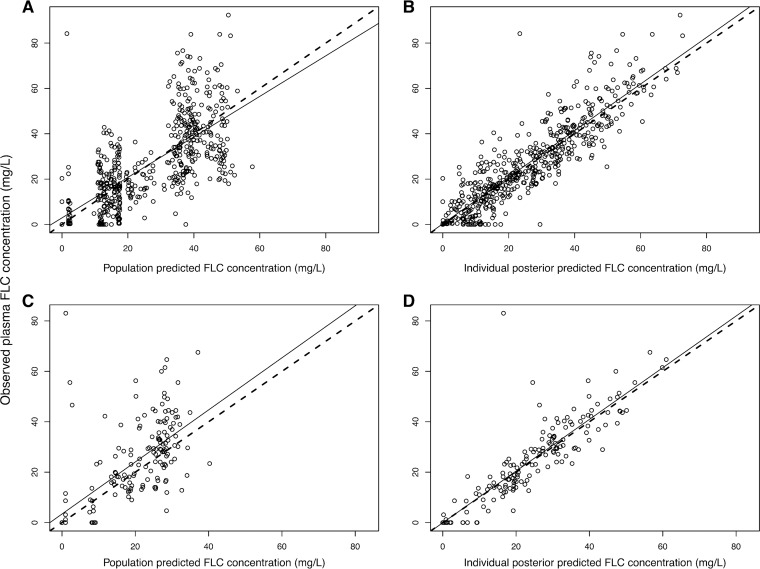
Scatter plots showing observed-versus-predicted values for the chosen population pharmacokinetic model after the Bayesian step. (A) Population predicted concentration of fluconazole in plasma. *R*^2^ = 0.49; intercept, 2.89 (95% CI, 0.51 to 5.27); slope, 0.89 (95% CI, 0.82 to 0.97). (B) Individual posterior predicted concentration of fluconazole in plasma. *R*^2^ = 0.80; intercept, 0.27 (95% CI, −1.08 to 1.62); slope, 1.03 (95% CI, 0.98 to 1.07). (C) Population predicted concentration of fluconazole in CSF. *R*^2^ = 0.46; intercept, 3.39 (95% CI, −0.09 to 6.87); slope, 1.03 (95% CI, 0.87 to 1.2). (D) Individual posterior predicted concentration of fluconazole in CSF. *R*^2^ = 0.81; intercept, −0.07 (95% CI, −1.97 to 1.84); slope, 1.03 (95% CI, 0.95 to 1.10). Circles, dashed lines, and solid lines represent individual observed-predicted data points, line of identity, and the linear regression of observed-predicted values, respectively. FLC, fluconazole; CI, confidence interval.

The final mathematical model was a linear model comprised of an absorption compartment, central compartment, peripheral compartment, and CSF compartment. The fit of the final model to the clinical data was acceptable. The mean parameter estimates better fitted the data than medians and were used to calculate Bayesian estimates of drug exposure for each individual patient. A linear regression of the observed-versus-predicted fluconazole concentrations in plasma after the Bayesian step was given by the following calculation: observed fluconazole concentration = 1.03 × predicted fluconazole concentration + 0.27 (*r*^2^ = 0.80). For the observed-versus-predicted fluconazole concentrations in CSF, the linear regression was given by the following: observed fluconazole concentration = 1.03 × predicted fluconazole concentration − 0.07 (*r*^2^ = 0.81) (Fig. 2 and Table 2). The mean weighted population bias values for fluconazole concentrations in plasma and CSF were 0.20 and −0.30, respectively. The bias-adjusted population imprecision values in plasma and CSF were 2.21 and 1.55, respectively. The population PK parameter estimates for the final model are shown in Table 3.

**TABLE 2:** Evaluation of the predictive performance of the considered and final models

Model and measured compartment^*a*^	Log likelihood	AIC^*b*^	Population bias	Population imprecision	Linear regression of observed-predicted values for each patient			*P* value^*d*^
					*R*^2^^*c*^	Intercept	Slope	
Model 1								
Plasma	−2,451	4,928	0.20	2.21	0.80	0.27	1.03	0.56
CSF			−0.30	1.55	0.81	−0.07	1.03	
Model 2								
Plasma	−2,413	4,854	0.36	2.38	0.80	0.01	1.03	
CSF			−0.41	1.81	0.80	0.89	1.01	

a Model 1 did not include any covariates. Model 2 incorporated a function to scale the volume of distribution in central compartment to patient weight.

b AIC, Akaike information criterion.

c Relative to the regression line fitted for the observed-versus-predicted values after the Bayesian step.

d Comparison of the joint distribution of population parameter values for each model.

**TABLE 3:** Population parameter estimates from the final 4-compartment pharmacokinetic model

Parameter^*a*^	Mean	Median	SD
*K_a_* (h^−1^)	8.78	1.73	11.98
SCL/*F* (liters/h)	0.72	0.65	0.24
*V*_c_/*F* (liters)	18.07	17.41	6.31
*K*_cp_ (h^−1^)	12.20	8.36	11.17
*K*_pc_ (h^−1^)	18.10	18.34	8.25
IC_gut_ (mg)	34.67	49.99	22.74
IC_central_ (mg)	35.86	49.98	19.67
IC_CNS_ (mg)	31.06	49.96	23.47
IC_peripheral_ (mg)	34.29	49.96	13.21
*K*_cs_ (h^−1^)	35.43	42.55	13.74
*K*_sc_ (h^−1^)	28.63	29.04	10.03
*V*_cns_/*F* (liters)	32.07	30.49	17.60

a SCL, clearance; *V_c_*, volume of distribution in central compartment; *F*, bioavailability; *K*_cp_, first-order rate constant from the central to peripheral compartment; *K*_pc_, first-order rate constant from the peripheral to central compartment; IC, initial condition in the respective compartment; *K*_cs_, first-order rate constant from the central to CNS compartment; *K*_sc_, first-order rate constant from CNS to central compartment; *V*_cns_, volume of distribution in CNS compartment.

### Covariate investigation.

Multivariate linear regression of each subject's covariates versus the Bayesian posterior parameter values revealed a weak relationship between patient weight and estimated volume of distribution (slope, 0.22; 95% confidence interval (CI) for the slope, −0.06 to 0.51; *P* value, 0.05). Incorporation of weight into the PK model was therefore explored. However, values for log likelihood, Akaike information criterion (AIC), and population bias and imprecision were comparable between the two models. The simple base model was therefore used to describe the data and for the subsequent simulations. The model comparisons and the fit to data are summarized in Table 2.

There was no relationship between the Bayesian estimates of clearance and volume and the covariate of either ethnicity or sex in the base model. The mean (95% CI) clearance was 0.74 liters/h (0.64 to 0.83 liters/h) and 0.71 liters/h (0.59 to 0.82 liters/h) for Vietnamese and Ugandan patients, respectively (*P* = 0.51). The mean (95% CI) volume was 16.88 liters (14.33 to 19.44 liters) and 19.44 liters (16.88 to 22.0 liters) for Vietnamese and Ugandan patients, respectively (*P* = 0.16). In males, the mean (95% CI) clearance was 0.79 liters/h (0.67 to 0.90 liters/h). In females, clearance was 0.66 liters/h (0.57 to 0.75 liters/h) (*P* = 0.09). In males, the mean (95% CI) volume was 18.07 liters (15.47 to 20.67 liters). In females, the mean volume was 18.07 liters (15.41 to 20.73 liters) (*P* = 0.97).

### Fluconazole penetration into the CSF.

There was large variability in the AUCs generated from each patient's posterior estimates. The 38 patients who received 800 mg of fluconazole q24h had a median (interquartile range [IQR]) AUC from 144 to 168 h after treatment initiation (AUC_144–168_) of 945.4 (799.2 to 1,139.8) mg · h/liter in plasma (AUC_plasma_) and 784.2 mg · h/liter (615.9 to 879.4) in CSF (AUC_CSF_). From these posterior estimates, the mean ratio of AUC_CSF_/AUC_plasma_ was 0.82 (standard deviation, 0.22).

**FIG 3:**
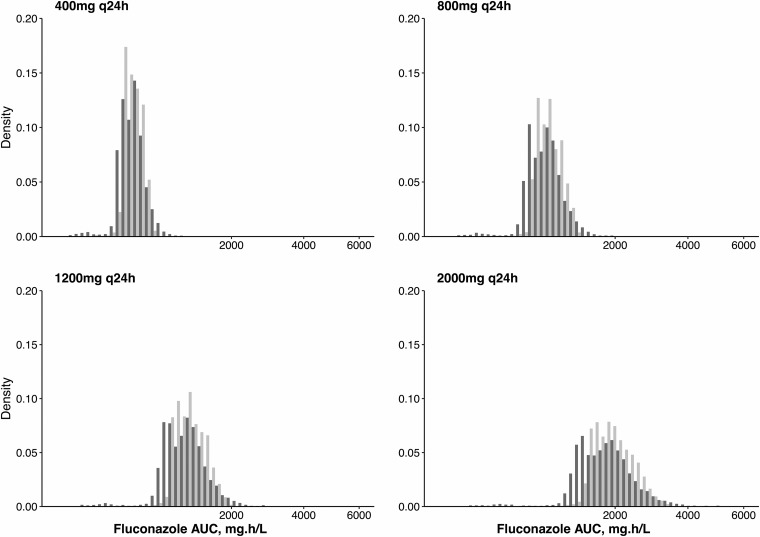
AUC distributions in 5,000 simulated patients at escalating fluconazole dosages. Light gray bars indicate simulated plasma AUC_144–168_. Dark gray bars indicate simulated CSF AUC_144–168_.

Monte Carlo simulation was used to estimate the distribution of drug exposure for dosages of 400 mg, 800 mg, 1,200 mg, and 2,000 mg q24h of fluconazole (Fig. 3). PK variability was marked, both in plasma and CSF. After administration of a dosage of 1,200 mg of fluconazole q24h, the median (IQR) simulated plasma AUC_144–168_ was 1,143.2 (988.4 to 1,378.0) mg · h/liter and the CSF AUC_144–168_ was 982.9 (781.0 to 1,185.9) mg · h/liter. The mean simulated ratio of AUC_CSF_/AUC_plasma_ was 0.89 (SD, 0.44).

### Probability of target attainment analysis.

**FIG 4:**
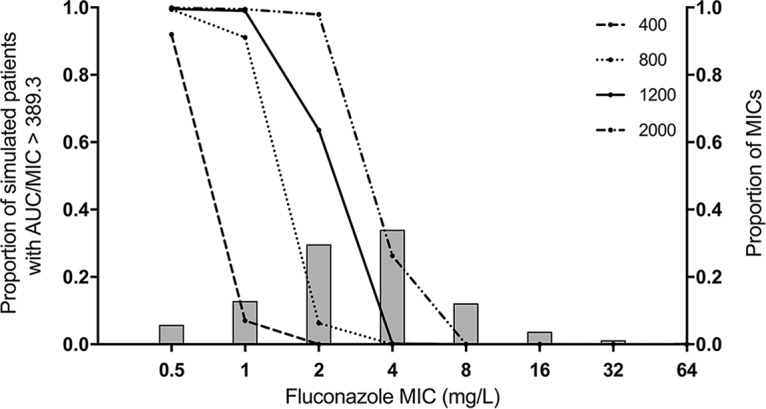
Probability of pharmacodynamic target attainment in plasma as a function of isolate MIC and fluconazole dosage. Each line represents the proportion of 5,000 simulated patients that achieve the PD target at the respective dosage (in milligrams) of fluconazole. The PD target was a plasma AUC/MIC ratio of ≥389.3. Bars show the proportion of WT strains of C. neoformans at the indicated MIC.

Monte Carlo simulation was used to predict the probability of achieving a total drug AUC/MIC ratio of ≥389.3 in plasma. This PD target was shown in a murine model of cryptococcal meningitis to be associated with a stasis endpoint (i.e., no net change in fungal density at the end of the experiment compared with that at treatment initiation) (11). Only 61% of simulated patients receiving 1,200 mg of fluconazole q24h achieved this PD target when the MIC for the infecting strain was 2.0 mg/liter. For MICs of ≥4.0 mg/liter, <1% of simulated patients administered 1,200 mg q24h achieved the PD target (Fig. 4).

### Meta-analysis of clinical outcome data.

A systematic review identified 163 relevant manuscripts, of which 11 were duplicates. After reviewing titles and abstracts, 28 studies were deemed potentially relevant for inclusion in the meta-analysis. Detailed examination of these studies resulted in the ultimate inclusion of 12 papers describing clinical outcomes from cryptococcal meningitis treated with fluconazole monotherapy. In total, 28 patients in 1 study received 200 mg of fluconazole q24h (20), 19 patients in 2 studies received 400 mg of fluconazole q24h (7, 21), 97 patients in 3 studies received 800 mg q24h (22–24), 113 patients in 4 studies received 1,200 mg q24h (8, 23–25), and 1 study described outcomes of 16 patients on 1,600 mg (24) and 8 patients on 2 g of fluconazole q24h (24). All included patients were HIV positive. Baseline characteristics and reported clinical outcomes are presented in Table 4.

**TABLE 4:** Baseline characteristics and clinical outcomes from trial data of fluconazole monotherapy by dosing regimen

Fluconazole dosage (mg)	Country	No. of patients	Age (yr)^*a*^	GCS <15 (%)	No. of CD4 cells/mm^3^^*a*^	CSF burden (log_10_ CFU/ml)	CSF sterility^*e*^	CSF sterility time point (wk)	2-wk mortality (%)^*e*^	10-wk mortality (%)^*e*^	Reference
200	Uganda	28	33 (23–50)^*b*^	43	73^*c*^		4/8 (50)	8	10/25 (40)	16/25 (64)	Mayanja-Kizza et al. (20)
400	USA	14	38 (2)^*c*^	0	44 (13)^*c*^	4^*d*^	6/14 (43)	10	NR	4/14 (29)	Larsen et al. (21)
	South Africa	5	39 (37–51)	60	41	5.53	NR^*f*^	NR	NR	3/4 (75)	Bicanic et al. (7)
800	Malawi	58	32 (29–39)	24	37 (11–58)		NR	NR	17/58 (29)	33/58 (57)	Rothe et al. (22)
	Uganda	30	35 (30–38)	33	7 (3–17)	5.7	NR	NR	11/30 (37)	18/30 (60)	Longley et al. (23)
	USA	9	35	100	8	4.8^*d*^	1/9 (11)	10	NR	8/9 (89)	Milefchik (24)
1,200	Malawi	47	35 (32–40)	24	36 (17–62)		NR	NR	16/47 (34)	26/47 (55)	Gaskell et al. (25)
	Uganda	30	33 (28–42)	60	14 (4–33)	5.9	NR	NR	6/27 (22)	13/27 (48)	Longley et al. (23)
	USA	16	40	100	36	3.5^*d*^	6/16 (37.5)	10	NR	10/16 (62.5)	Milefchik et al. (24)
	Malawi	20	36.5 (27–71)^*b*^	40	25 (1–66)^*b*^	5.30	1/20 (5)	2	7/19 (37)	11/19 (58)	Nussbaum et al. (8)
1,600	USA	16	35	100	33	3^*d*^	10/16 (62.5)	10	NR	6/16 (37.5)	Milefchik et al. (24)
2,000	USA	8	36	100	35	2.4^*d*^	5/8 (62.5)	10	NR	3/8 (37.5)	Milefchik et al. (24)

a Median (interquartile rage) unless otherwise specified.

b Values in parentheses are range.

c Value is mean (standard error).

d Extrapolated from cryptococcal antigen titer.

e Fraction (%) of patients.

f NR, not reported.

**FIG 5:**
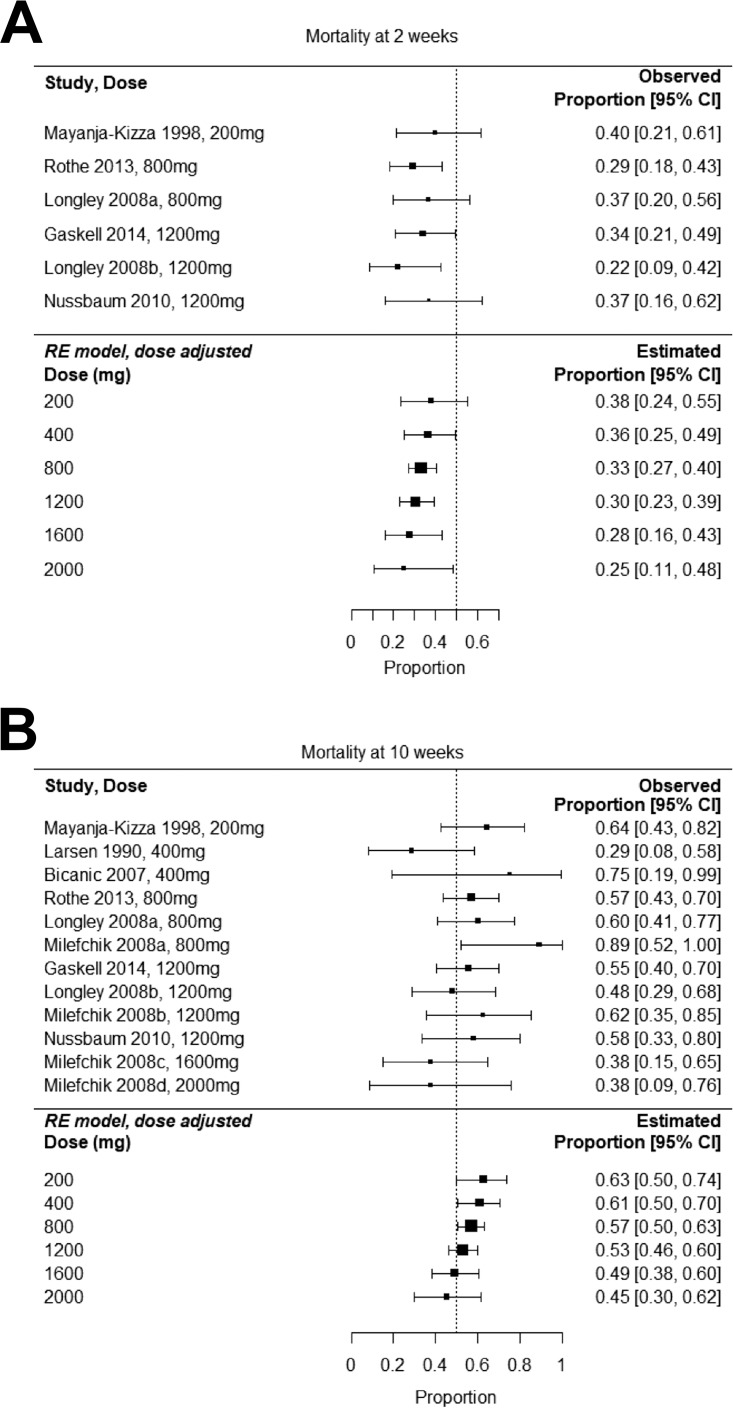
Meta-analysis of clinical trials of fluconazole monotherapy showing dose-adjusted effects on 2-week mortality (A) and 10-week mortality (B). Right-hand columns provide observed and estimated proportions of patients dead at the indicated time.

The final model suggests that the combination of dose and baseline fungal burden explains the total heterogeneity in the estimated proportion of patients with sterile CSF after 10 weeks of treatment (*P* value for residual heterogeneity, 0.64). However, there was not a significant relationship between dose and CSF sterility at 8 to 10 weeks (*P* value, 0.45). After adjustment for dose, the test for residual heterogeneity in both 2- and 10-week mortality was not significant (*P* values, 0.70 and 0.22, respectively), indicating that dose alone adequately explained total heterogeneity in mortality outcomes at both time points. For both 2- and 10-week mortality outcomes, there was a nonsignificant trend toward reduced mortality with escalating dosage (Fig. 5).

## DISCUSSION

Fluconazole is the only drug available for induction therapy for cryptococcal meningitis in many regions of the world where the incidence of disease is highest. An accumulating body of evidence suggests that fluconazole is a suboptimal agent for this indication (26). While this has long been recognized, an explanation for the relatively poor efficacy of fluconazole is absent. This study presents a uniquely comprehensive clinical data set describing the PK of fluconazole. It provides robust estimates of CNS penetration and the variability of those estimates. A high degree of CNS partitioning has been observed in previous clinical studies with fluconazole (19, 27). Distribution into the CNS is facilitated by low molecular weight, low protein binding, and moderate lipophilicity (15, 28). Fluconazole has proven activity against Cryptococcus neoformans (29, 30). This study provides a further understanding as to why, despite these attributes, fluconazole is inferior to amphotericin B deoxycholate as an agent for induction monotherapy for cryptococcal meningitis (6–8).

In contrast to previous studies of fluconazole PK (31–33), our data do not suggest a significant relationship between fluconazole clearance and creatinine clearance nor between patient weight and volume of distribution. The reason for this is not immediately clear but may relate to the relatively narrow range of creatinine clearance in our population and the fact that the vast majority of patients in our cohort had low body weight, with the range of this covariate also being relatively narrow.

The PK model suggests that current regimens of fluconazole are inadequate for induction therapy for cryptococcal meningitis. This has routinely been ascribed to the overly simplistic notion that fluconazole is a fungistatic agent. Our analyses provide further insight into the limitations of this drug. Previous estimates of fluconazole CNS/plasma partition ratios have ranged from 0.52 to 1.33 (18, 19, 27, 34). We have extended these estimates by rigorously quantifying the marked variability in the CSF PK. This variability has consequences at both microbiological and clinical levels. Suboptimal exposure of fluconazole promotes the expansion of intrinsically resistant cryptococcal subpopulations present at the initiation of therapy (35). In addition, the evolution of C. neoformans during therapy to become increasingly triazole resistant has been demonstrated in clinical studies (36, 37). To be clinically effective, adequate concentrations of drug must be present at the site of infection for long enough to exert an antimicrobial effect on both susceptible and resistant subpopulations. The present analysis demonstrates the challenges in achieving that aim.

At the recommended fluconazole dosage of 1,200 mg q24h, the probability of PD target attainment (PTA) bisects the MIC distribution for wild-type (WT) C. neoformans isolates. This is consistent with the findings of Sudan et al. (11). Approximately half of patients will fail therapy because they are not able to generate the drug exposure required to prevent progressive fungal growth. Since clinical PK-PD targets are not available for fluconazole in cryptococcal meningitis, we have used a target derived from a murine study (11). This assumes that CNS partitioning is the same in mice and humans. The cerebrum/plasma AUC ratio in the murine study was 46.9% (11). It is conceivable that this is in keeping with our CSF/plasma AUC ratio of 82% though clearly it would be preferable to have clinical PK-PD targets defined. Nevertheless, our PTA analysis is supported by the 53% 10-week mortality outcomes for patients receiving 1,200 mg of fluconazole q24h, estimated in the meta-analysis. Importantly, such PTA analyses are based on an AUC/MIC of 389.3, which is more than an order of magnitude greater than the AUC/MIC ratio required for Candida albicans (12).

Progressive escalation of the dosage of fluconazole is not likely to be an effective strategy for improving cryptococcal meningitis induction therapy. The drug exposure required to reliably treat isolates with MICs of ≥4.0 mg/liter is difficult to achieve and potentially toxic. Our meta-analysis suggests that escalating dosages of fluconazole do not increase the proportion of patients with sterile CSF at 10 weeks. Dosages of 2,000 mg q24h do not appear to significantly improve 10-week mortality outcomes in comparison to a dose of 1,200 mg q24h. The AIDS Clinical Trials Group (ACTG) study (https://clinicaltrials.gov/show/NCT00885703) is investigating the use of higher dosages of fluconazole (1,600 mg and 2,000 mg q24h) for the treatment of cryptococcal meningitis in HIV-infected individuals, and results are pending. The addition of flucytosine to high-dose fluconazole (≥1,200 mg q24h) for cryptococcal meningitis increases antifungal activity and improves mortality outcomes (8, 24), suggesting that combination therapy is required to optimize antifungal activity in fluconazole-containing regimens.

In summary, this study provides part of the pharmacodynamic rationale for the long-recognized fact that fluconazole monotherapy is an ineffective induction regimen for cryptococcal meningitis. We have developed a fluconazole population PK model that suggests that approximately half of patients with cryptococcal meningitis caused by WT strains of C. neoformans will be undertreated by currently recommended dosages of fluconazole for induction therapy. In doing so, we have addressed a knowledge gap regarding the reason for the inferiority of this drug for cryptococcal meningitis. There is a pressing need for improved provision of affordable combination treatments and development of more effective drugs.

## MATERIALS AND METHODS

### Clinical pharmacokinetic studies.

Patients from whom plasma and CSF samples were obtained for this PK study have been described previously (38). Briefly, adult patients (*n* = 3) were initially recruited from a multicenter randomized controlled trial of adjuvant dexamethasone in HIV-associated cryptococcal meningitis. The trial is reported elsewhere (International Standard Registered Clinical Number 59144167) (38). Following the early cessation of this trial, patients were recruited from a prospective descriptive study at the same sites (*n* = 40). Study sites were The Hospital for Tropical Diseases in Ho Chi Minh City, Vietnam, and Masaka General Hospital, Uganda. The study protocols were approved by the relevant institutional review boards and regulatory authorities at each trial site and by the Oxford University Tropical Research Ethics Committee.

Fluconazole was administered orally. In cases where the conscious level of the patient did not enable oral administration, fluconazole was administered via nasogastric tube. The majority of patients received 800 mg of fluconazole q24h. Two patients received one-off doses of 400 mg q24h. Two received one-off doses of 600 mg q24h. One patient's regimen of 800 mg of fluconazole q24h was escalated to 1,200 mg q24h for 6 days from day 8 of treatment. All patients received combination therapy with amphotericin B deoxycholate at 1 mg per kg infused over 5 to 6 h.

### Measurement of fluconazole concentrations.

Fluconazole concentrations were measured using a validated liquid chromatography-tandem mass spectrometry (LC-MS/MS) methodology (1260 Agilent UPLC [ultra-performance liquid chromatograph] coupled to an Agilent 6420 Triple Quad mass spectrometer; Agilent Technologies UK, Ltd., Cheshire, United Kingdom). Briefly, fluconazole was extracted by protein precipitation; 300 μl of cold methanol containing the internal standard fluconazole-D4 at 0.625 mg/liter (TRC, Canada) was added to 10 μl of sample (plasma or CSF). The solution was vortex mixed for 5 s and filtered through a Sirocco precipitation plate (Waters, Ltd., Cheshire, United Kingdom). Supernatant (150 μl) was transferred to a 96-well auto sampler plate, and 3 μl was injected on an Agilent Zorbax C_18_ Rapid Resolution High Definition (RRHD) column (2.1 by 50 mm; particle size, 1.8 μm) (Agilent Technologies UK, Ltd., Cheshire, United Kingdom).

Chromatographic separation was achieved using a gradient consisting of 70% A/30% B (0.1% formic acid in water as mobile phase A and 0.1% formic acid in methanol as mobile phase B). The organic phase was increased to 100% over 90 s, with an additional 90 s of equilibration.

The mass spectrometer was operated in multiple-reaction-monitoring scan mode in positive polarity. The precursor ions were 307.11 *m/z* and 311.1 *m/z* for fluconazole and the internal standard, respectively. The product ions for fluconazole were 220.1 *m/z* and 238.1 *m/z*; for the internal standard the product ions were 223.2 *m/z* and 242.1 *m/z*. The source parameters were set as follows: capillary voltage, 4,000 V; gas temperature, 300°C; and nebulizer gas, 15 lb/in^2^.

The standard curve for fluconazole encompassed the concentration range of 1 to 120 mg/liter and was constructed using blank matrix. The limit of quantitation was 1 mg/liter. In plasma, the intraday coefficient of variation (CV) was <3.4%, and the interday CV was <6.7% over the concentration range of 1 to 90 mg/liter. In CSF, the intraday CV was <5.2%, and the interday CV was <5.3% over the same concentration range.

### Population pharmacokinetic modeling.

The concentration-time data for fluconazole in plasma and CSF were analyzed using the nonparametric adaptive grid (NPAG) algorithm of the program Pmetrics (39), version 1.5.0, for the R statistical package, version 3.1.1. The initial PK mathematical model fitted to the data contained four compartments and took the following form:

(1) $$\frac{dX(1)}{dt}=-K_{a}\times X(1)$$

(2) $$\frac{dX(2)}{dt}=K_{a}\times X(1)-\left(K_{\text{cp}}+K_{\text{cs}}+\frac{\text{SCL}}{V}\right)\times X(2)+K_{\text{sc}}\times X(3)+K_{\text{pc}}\times X(4)$$

(3) $$\frac{dX(3)}{dt}=K_{\text{cs}}\times X(2)-K_{\text{sc}}\times X(3)$$

(4) $$\frac{dX(4)}{dt}=K_{\text{cp}}\times X(2)-K_{\text{pc}}\times X(4)$$

(5) Y(1)=X(2)/V

(6) $$Y(2)=X(3)/V_{\text{cns}}$$

where equations 1, 2, 3, and 4 describe the rate of change in amount of drug in milligrams in the gut, central, CSF, and peripheral compartments, respectively. *K_a_* is the absorption rate constant from the gut to the central compartment. *X*(*1*), *X*(*2*), *X*(*3*), and *X*(*4*) are the amounts of fluconazole (in milligrams) in the gut, central (c), CSF (s) and peripheral compartments (p), respectively. *K*_cp_, *K*_pc_, *K*_cs_, and *K*_sc_ represent first-order transfer constants connecting the various compartments. SCL is the first-order clearance of drug (liters/hour) from the central compartment. *V* is the volume of the central compartment. The CSF compartment [*X*(*3*)] has an apparent CSF volume (*V*_cns_), given in liters. Equations 5 and 6 are the output equations describing fluconazole levels in the central and CSF compartments, respectively. The output in each compartment is denoted *Y*.

Model error was attributed separately to process noise (including errors in sampling times or dosing) and assay variance. Process noise was modeled using lambda, an additive error term. The data were weighted by the inverse of the estimated assay variance.

The data for some patients indicated that they had taken fluconazole at an undocumented time prior to study enrollment since there was detectable drug in the first PK sample. To accommodate this, nonzero initial conditions of all four compartments were estimated in the structural model. A switch was coded whereby the parameterized estimate of each initial condition was multiplied by a binary covariate equal to 1 when fluconazole was detected in the first PK sample or by 0 when no fluconazole was detected in the first PK sample.

### Population pharmacokinetic covariate screening.

The impacts of patient weight, BMI, sex, ethnicity, and baseline eGFR on the PK of fluconazole were investigated. Bidirectional stepwise multivariate linear regression was employed to assess the relationship between each covariate and the Bayesian estimates for volume of distribution and clearance from the central compartment from the standard population PK model. Covariates that were retained with significant multivariate *P* values (≤0.05) in the regression model were explored individually. The relationship between retained continuous covariates and Bayesian estimates of PK parameters was explored using univariate linear regression. The difference between Bayesian estimates of volume and clearance according to categorical covariates (sex and ethnicity) was compared using a Mann-Whitney test.

### Population pharmacokinetic model diagnostics.

The fit of the model to the data was assessed by visual inspection of diagnostic scatterplots displaying observed-versus-predicted values before and after the Bayesian step. Linear regression was performed, and the coefficient of determination, intercept, and regression slope were noted for each model. In addition, the log-likelihood value, Akaike information criterion (AIC), mean weighted error (a measure of bias), and bias-adjusted, mean weighted squared error (a measure of precision) were calculated and compared for each model.

### Monte Carlo simulation and calculation of probability of target attainment.

Monte Carlo simulation (*n* = 5,000) was performed in Pmetrics (39). The support points from the final joint density were used. For the simulations, the initial conditions of all compartments were defaulted to zero. Fluconazole was administered at a range of dosages: 400 mg q24h, 800 mg q24h, 1,200 mg q24h, and 2,000 mg q24h. The plasma and CSF AUC values for fluconazole were calculated using trapezoidal approximation after the sixth dose, from 144 to 168 h after treatment initiation.

Wild-type fluconazole MIC data were obtained from a previously published collection of 5,733 C. neoformans isolates estimated using Clinical and Laboratory Standards Institute (CLSI) methodology (40). The modal MIC was 4 mg/liter (1,629 of 5,733 strains; 28%). Almost half of strains had MICs of ≥4 mg/liter (2,834 of 5,733 strains; 49%). The epidemiological cutoff value for C. neoformans versus fluconazole was 8 mg/liter. This collection of strains included molecular types VNI to VNIV, and the patterns of MIC distribution were comparable across all molecular types (40). The proportion of simulated patients that would achieve a previously published plasma AUC/MIC target of 389.3 was determined. This target was defined as the magnitude of drug exposure required for fungal stasis (defined as prevention of progressive fungal growth) in a murine study that employed CLSI methodology (11). To our knowledge, no CSF PK/PD target has been defined in preclinical or clinical studies of fluconazole for cryptococcal meningitis. In the present study, the probability of attaining this plasma PK/PD target was examined at each simulated fluconazole dose.

### Meta-analysis of clinical outcome data.

The AUC/MIC target used in the probability of target attainment analysis was derived from murine studies. To enhance clinical relevance, we sought PD data from humans. The PD data from patients in the present PK study are confounded by the coadministration of amphotericin B deoxycholate. For this reason, a search for clinical trials of fluconazole monotherapy for cryptococcal meningitis was performed. The electronic databases Pubmed and Medline were searched on 31 January 2018 using the terms “fluconazole” and “cryptococcal meningitis.” Preclinical studies and case reports were excluded. To reduce potential heterogeneity, only studies of HIV-positive participants were included in the meta-analysis. Baseline variables were chosen *a priori* for extraction from the studies if they had previously been determined to have a significant impact on clinical outcome. These were mental status, CSF fungal burden, and patient age (6, 41). Where it was not reported, baseline CSF fungal burden was extrapolated from CSF cryptococcal antigen titer according to a correlation published by Jarvis et al. (6).

For consistency with the literature, we collected data on clinical outcomes commonly presented in cryptococcal meningitis trials: CSF sterility at 8 to 10 weeks, 2-week mortality, and 10-week mortality. Mixed-effects meta-analysis adjusted for fluconazole dosage was performed. Fungal burden in CSF, CD4 count, and proportion of patients with reduced Glasgow coma score (GCS) at baseline were explored to assess the degree to which these modifiers accounted for interstudy heterogeneity in clinical outcome. The mixed-effects model took the form: θ_*i*_ = β_0_ + β_1_*Z_i_*_1_ + … + β_1_*Z_ij_* + *u_i_*, where θ_*i*_ is the corresponding (unknown) true effect of the *i*th study, *Z_ij_* is the value of the *j*th moderator variable for the *i*th study with corresponding model coefficients β, and *u_i_* are study-specific random effects such that *u_i_* ∼ *N*(0,τ^2^). Here, *N* indicates that the random effects are normally distributed, 0 is the mean of the random effects, and τ^2^ denotes the amount of residual heterogeneity, estimated using the DerSimonian-Laird estimator (42). Additional model parameters were estimated via weighted least squares with weights relative to the estimated τ^2^. The null hypothesis *H*_0_:τ^2^ = 0 was tested using Cochran's Q-test, and model parameters were tested with the Wald-type test statistic.
